# High performing primary health care organizations from patient perspective: a qualitative study in China

**DOI:** 10.1186/s41256-023-00315-0

**Published:** 2023-08-07

**Authors:** Wenhua Wang, Jinnan Zhang, Katya Loban, Xiaolin Wei

**Affiliations:** 1 School of Public Policy and Administration, Xi’an Jiaotong University, Xi’an, People’s Republic of China; 2 Research Institute of the McGill University Health Centre, McGill University, Montreal, Canada; 3 Dalla Lana School of Public Health, University of Toronto, Toronto, Canada

**Keywords:** China, Primary health care, Organizational characteristics, Health policy, Patient preference, Qualitative research

## Abstract

**Background:**

There is a global call to build people-centred primary health care (PHC) systems. Previous evidence suggests that without organization-level reform efforts, the full potential of policy reforms may be limited. This study aimed to generate a profile of high performing PHC organizations from the perspective of patients.

**Methods:**

We conducted semi-structured interviews with 58 PHC users from six provinces (Shandong, Zhejiang, Shaanxi, Henan, Shanxi, Heilongjiang) in China using purposive and snowball sampling techniques. Transcription was completed by trained research assistants through listening to the recordings of the interviews and summarizing them in English by 30-s segments to generate the narrative summary. Informed by the Classification System of PHC Organizational Attributes, thematic analysis aimed to identify domains and attributes of high performing PHC organizations.

**Results:**

A profile of a high performing PHC organization with five domains and 14 attributes was generated. The five domains included: (1) organizational resources including medical equipment, human and information resource; (2) service provision and clinical practice including practice scope, internal integration and external integration; (3) general features including location, environment and ownership; (4) quality and cost; and (5) organizational structure including continuous learning mechanism, administrative structure and governance.

**Conclusions:**

A five-domain profile of high performing PHC organizations from the perspective of Chinese PHC users was generated. Organizational resources, service delivery and clinical practices were most valued by the participants. Meanwhile, the participants also had strong expectation of geographical accessibility, high quality of care as well as efficient organizational structure. These organizational elements should be reflected in further reform efforts in order to build high performing PHC organizations.

## Background

Primary health care (PHC), as defined by the World Health Organization (WHO), is an approach to health that aims to ensure the highest possible level of health and wellbeing and equitable distribution by focusing on people´s needs and preferences as early as possible along the continuum from health promotion and disease prevention to treatment, rehabilitation, and palliative care, and as close as feasible to people’s everyday environment [1]. As a necessary foundation of universal health coverage (UHC) and the Sustainable Development Goals (SDGs), which is an ambitious development agenda aimed at improving the health and well-being of all people, PHC incorporates three interrelated and synergistic components: multisectoral policy and action, integrated primary care and essential public health services, and empowered people and community [1]. Over the past 4 decades, considerable evidence has been generated demonstrating the significant contribution of PHC to better health outcomes, improved equity, increased health security and cost-efficiency [2].

Based on the achievements of previous PHC reform efforts, the WHO has recently issued an operational framework identifying 4 core strategic levers and 10 operational levers to guide further global PHC-oriented system strengthening in the twenty-first century [2]. Whilst these levers are based on existing evidence and global reform experiences, there is still a general lack of knowledge about how to best apply or integrate these various strategies and approaches in practice for frontline PHC organizations. Without organization-level reform efforts, the full potential of policy reforms may be limited [3, 4]. As part of broader health systems at the district and national levels, PHC organizations play a foundational role in bridging the three components of PHC. Previous studies suggest that the success of various strategies or interventions depends on the organizational context in which they are implemented [3, 5]. However, there is limited information on the organizational context of high performing PHC organizations, especially in low-and-middle income countries.

China is currently reorienting the health service delivery system towards building a PHC-based People-Centred Integrated Care model as suggested by the WHO and the World Bank [6]. Over the past decade, China has undertaken reform efforts to build high performing PHC organizations. The government has made significant investments in building well-equipped primary care organizations through increasing financial subsidies, infrastructure building and allocating medical equipment, as well as training more primary care professionals [7–9]. In recent years, national guidelines and standards have been issued to guide the transformation of primary care organization into high performing entities. The guidelines focused on such areas as internal organizational structure, scope of practice, service delivery approach, and performance evaluation [7, 10–15]. To guide further reform efforts, deeper knowledge of organizational context of high performing PHC organizations is required. This study aimed to generate a clear profile of high performing PHC organizations from the perspective of patients, in order to inform subsequent reform efforts.

## Methods

### Setting and study design

A qualitative descriptive study using semi-structured interviews was conducted in six provinces in China. The study covered the government designated eastern region (Jinan City in Shandong Province and Hangzhou City in Zhejiang Province); central region (Luoyang City in Henan Province, Taiyuan City in Shanxi Province and Harbin City in Heilongjiang Province); and western region (Xi’an City in Shaanxi Province).

The eligible criteria for participation were: 18–80 years old, primary care user, no cognitive impairment, and having visited a PHC organization during the past 12 months. The details of recruitment can be found in a previous study [16]. Finally, 58 interviewees completed the interview.

### Interview guide development

The interview guide mainly included the following information: general information on the participants; participants’ health and care seeking behaviours; and what constitutes “good primary health care organizations” from the participants’ point of view. Details of guide development can be found in a previous study conducted by our team [16].

### Data collection and processing

After obtaining informed consent from participants, we conducted semi-structured interviews from April to December 2021. Interviews were conducted in Mandarin by trained research assistants (RAs) who are graduate students in a health policy and management program and have extensive experience in qualitative health research. During the interview process, the interviewers first asked participants to describe their health care seeking experiences during the past year and helped them to recall information relating to those visits. Then, the interviewers encouraged the participants to share in their own words their opinions and expectations for high performing primary health care organizations [17]. The interviews were conducted face-to-face, one-on-one and lasted between 30 and 60 min. With the consent of the interviewees, all interviews were audio-recorded [18]. Interview recordings were uploaded into NVivo12 software.

### Data analysis

The data were analysed using thematic analysis [19]. We used the Classification System of PHC Organizational Attributes developed by the Institut national de santé publique du Québec (INSPQ) [20] as a guiding framework. Our analysis was predominantly descriptive, combining deductive and inductive approaches to allow original themes to emerge [21]. First, two trained RAs listened to the recordings of the interviews and summarized them in English for each 30-s segment. Parallel to that, the RAs captured key phrases and made notes about the behaviour of the interviewees, such as hesitations, lack of comfort, refusal to answer or tangents. This data transcription approach has been tested and used by other qualitative researchers [22]. Next, thematic analysis was performed. The two RAs worked independently to generate codes. They met regularly to compare coding, resolve differences, and refine code definitions and structure. Similar codes were grouped into categories and subsequently mapped to the domains from the framework. Emerging findings were discussed with the research team. Once the codebook was finalized, one RA continued to code the remaining narrative summaries [23].

### Trustworthiness

Several strategies were used to ensure trustworthiness in our research [24]. Credibility was enhanced through iterative questioning and debriefing sessions among research team members, as well as peer scrutiny by researchers from China and Canada. The detailed description of methods was provided to ensure dependability. Finally, fields notes, the products of the research and detailed notes capturing changes in the research process and interview guides were kept as an “audit trail” to support confirmability.

## Results

### Participant characteristics

Table 1 provides a summary of the characteristics of 58 participants. Half of the participants were female and half had received college or above education. 20% of the participants were over 60 years of age, and a quarter had one or more chronic diseases. For health insurance coverage, 70% were covered by the urban employee basic medical insurance, which is an insurance program for people who have or had a job, and the rest were covered by urban and rural resident basic medical insurance, which covers the unemployed, students and children.

**Table 1:** Demographics of 58 participants from six provinces

Characteristic	No. (%)
Age, mean (SD), years	45 (14.3)
< 60	47 (81.0)
≥ 60	11 (19.0)
Sex	
Male	29 (50.0)
Female	29 (50.0)
Education level	
Primary school or below	4 (6.9)
Junior school	10 (17.2)
High school or technical secondary school	14 (24.1)
Undergraduate or junior college	20 (34.5)
Master’s degree or higher	10 (17.2)
Medical insurance	
Employee basic medical insurance	41 (70.7)
Resident basic medical insurance	17 (29.3)
Chronic diseases status^a^	
Yes	14 (24.1)
No	44 (75.9)

^a^Chronic diseases here only included diabetes and/or hypertension

### Patient views of high performing PHC organizations

As shown in Table 2, five domains including 14 attributes were generated. The five domains were (in order of importance, from most frequently to least frequently mentioned): (1) Organizational Resources, (2) Service Provision and Clinical Practice, (3) General Features, (4) Quality and Cost, and (5) Organizational Structure.

**Table 2:** Five domains, 14 attributes and illustrative quotes from qualitative interviews with 58 PHC patients

Domains	Attributes	Exemplar quotes
Organizational resources (55)	Medical equipment and medicines (49)	“It is sufficient for CHCs to have basic medical equipment to do general tests.” (HRB5)
	Human resources (40)	“Patients can use their cell phones to register and pick up test results from the self-service machines.” (HZ3)
	Health information resources (12)	“A CHC should be equipped with a certain number of general practitioners.”
Service provision and clinical practice (48)	Practice scope (39)	“CHCs can treat or relieve common illnesses such as colds and fevers and minor bruises and injuries.” (JN1) “The CHCs can inform residents regarding health check-ups, such as urine and stool tests, blood tests, blood pressure measurements, etc.” (TY2)
	Internal integration (32)	“A set of procedures for registration, consultation, infusion, drug purchase, etc. should be reasonably configured and smoothly connected.” (TY2)
	External integration (16)	“When the CHCs can't handle it, they can proactively help the patient access the hospital and transfer the patient's electronic medical record to the hospital.” (TY6)
General features (48)	Location (43)	“A CHC should be closer to home, so that it is more convenient for me.” (HZ3)
	Environment (25)	“As a minimum, CHCs should maintain a clean environment.” (TY7)
	Ownership (17)	“CHCs should be public and managed by the government.” (TY2)
Quality and cost (39)	Quality (37)	“Patients’ treatment must be effective.” (HRB2) “I hope the experience of visiting the CHC is improved.” (LY5) “I also have expectations regarding medical safety. Medical errors should be avoided in a good CHC.” (LY1)
	Cost (20)	“For minor diseases such as colds and fevers, it is unreasonable for medical expenses to exceed 50 yuan. If the illness is serious, it should probably be within 100 yuan. The cost cannot be too high.” (LY6)
Organizational structure (34)	Continuous learning mechanism (20)	“PHC professionals should be continuously deepening their clinical expertise and tailoring their attitude and communication style to the patients.” (TY7)
	Administrative structure (15)	“The rules and regulations of CHCs should be detailed and made transparent.” (HRB4)
	Governance (11)	“It is inconvenient that health insurance cards from other areas cannot be used at the CHCs here.” (HZ8)

The brackets identify the number of participants expecting this domain/attributes, and the city of the representative quote: LY is Luoyang; HZ is Hangzhou; XA is Xi’an; JN is Jinan; TY is Taiyuan; HRB is Harbin. CHC stands for Community Health Center

For Organizational Resources, the common patient expectation is that a high performing PHC organization should have sufficient technical resources, including medical equipment meeting general needs, health information systems (e.g., well-functioning electronic medical record system), and human resources including a certain number of general practitioners.

For Service Provision and Clinical Practice, the patients expected a high performing PHC organization to provide comprehensive health services through an integrated approach. For integration within the organization, simple and smooth consultation process was the primary expectation. For integration with other organizations, the patients desired that PHC organizations be closely connected to hospitals through an efficient referral system, particularly effective clinical communication and sharing of patient electronic medical records.

For General Features, the patients expected a PHC organization to be close to home, to be a clean and tidy environment, and managed by the government. For the Quality and Cost domain, the patients’ common expectation is that the care that they receive should be effective and safe, with reasonable prescriptions, positive care experiences and lower costs. For Organizational Structure, the patients expect a continuous learning mechanism, including continuing training of PHC professionals to improve their clinical skills and communication abilities. They also expect that high performing PHC organizations have an efficient management team, clear role definition, and transparency. In addition, they also hope that PHC organizations follow the fee schedule and insurance policy issued by the government and undergo regular performance assessment by the government.

## Discussion

This study aims to generate a profile of high performing PHC organizations from the perspective of patients in China. Based on a qualitative study, we found that the profile of high performing PHC organizations include five domains and 14 attributes. From patients’ perspective, high performing PHC organizations should be near home with a comfortable environment and sufficient organizational resources; have efficient service delivery and clinical practice approach; and provide high-quality and low-cost comprehensive services, through an integrated approach supported by an efficient organizational structure.

The provision of basic medical services and public health services are the two main functions of PHC organizations as required by the Chinese government. As one key component of a PHC organization, organizational resources, including equipment, human resources and information systems, were valued by most participants in this study. The participants argued that basic medical equipment should be adequately provided in high performing PHC organizations—a finding that is consistent with the conclusions from a study in Germany [25]. From the perspective of our participants, a PHC organization with conventional medical equipment could perform its functions well. It is not necessary to invest into more advanced medical equipment, which might lead to resource waste and higher costs for the patients. Meanwhile, studies have also shown that healthcare professionals in well-equipped health organizations may not necessarily provide high quality care [26, 27]. As a result of significant investment in PHC during the past decade, adequate basic equipment is now available in most PHC organizations in China [28].

However, challenges remain in relation to health information system building and training enough general practitioners in China to better meet patient expectations in these areas. Health information systems are suffering from inadequate integration in China, such as the difficulty in linking the Electronic Medical Record Systems in PHC organizations with the systems used in public hospitals [9]. The number of qualified general practitioners remains insufficient and unequally distributed geographically. In 2020, the average number of general practitioners in China was 2.90 per 10,000 population (3.43 in eastern region, 2.53 in central region, and 2.47 in western region) [29]. These proportions are much lower than those in some developed countries, with 12.30 in Canada, 10.19 in Australia and 7.76 in the UK [30].

Among the three attributes of general features, short distance between a PHC organization and home was valued most by the participants. Similarly, previous studies in China and other countries have shown that distance is a predictor of patients’ choice of healthcare providers [25, 31, 32]. As places for treating many minor acute conditions, PHC organizations should be near home to ensure speedy treatment. In addition, there is substantial evidence of a distance-decay association whereby increased patient-provider distance impacts patient access to health services and health outcomes [33, 34]. In 2017, a national policy was issued to ensure that each Chinese resident reaches the nearest healthcare organization within 30 min in order to increase service accessibility [35]. Recent Chinese data show that the percentage of residents who could reach the nearest healthcare facility in less than 30 min increased from 95.8% in 2008 to 98.7% in 2018 [29].

Regarding the service delivery and clinical practice domain, the participants expressed strong expectations regarding a broader scope of services provided in an integrated manner. For internal integration, the consultation process was expected to be simple and smooth, specifically including front desk guides, clear signage, and reasonable department locations. The studies from the UK, US and Denmark also revealed that smooth consultation process could reduce waiting time and improve service efficiency [36]. In China, efficient service process requirements were already set out in a national 2019 guideline for PHC organization service capacity evaluation [12]. However, the policy seems to be poorly implemented by frontline PHC organizations, revealing the lack of collaboration between internal departments and cumbersome patient flow processes [37].

For external integration, PHC organizations were expected to maintain close connections with other health care providers through efficient referral systems, and to facilitate clinical communication—a finding that resonates with conclusions from a US study involving PHC patients [31, 38]. Prior studies suggest that efficient referral systems could prevent unnecessary financial and health losses for patients when the PHC organization capacity is limited [32, 38, 39]. China is currently examining different types of integrated care models, mainly focusing on reforming governance structures, payment methods, and care delivery models [40]. However, the referral system has not been well implemented [14]. Cross-referrals from either the PHC organizations or hospitals are limited due to profitability considerations [6]. In addition, cross-referrals are currently not supported due to a lack of integrated health information systems.

Quality of care, including effectiveness, safety, and patient experience, were given higher priority by participants than cost. These characteristics are recognized globally as important elements of a health system output [41]. Supported by the national essential medicine and medical insurance policy reform, out-of-pocket costs for PHC services have been greatly reduced in China. Yet the current quality of care in PHC organizations remains a challenge, with frequent diagnostic and treatment errors and overuse of antibiotics [42–45]. To improve the quality of PHC services, China could develop a PHC organization accreditation system based on the 2019 PHC Organization Service Capacity Evaluation Guidelines and the 2020 PHC Performance Evaluation Guidelines [12, 15].

Another important finding was that nearly 60% of our study participants desired high performing PHC organizations to have efficient organizational structures, particularly the continuous learning mechanism, transparent and efficient management, positive organizational culture and regular performance monitoring. There is growing evidence from other countries to support the positive impact of these organizational characteristics on performance within health care organizations [46–49]. These organizational variables represent potential management reform levers that could be used to better meet patients’ expectations and improve service performance. As China is moving towards building high quality primary care system, further studies are needed to examine the effect of these features on quality of care in Chinese PHC settings.

This study captured patient priorities regarding high performing PHC organizations in China and have some implications for practice and policy. The five domains and 14 attributes can be used by policy makers and PHC managers to guide future reform and restructuring efforts and to identify gaps in the organization of services. Based on the analysis of patient expectations and recent policy reform efforts in PHC, we found that China has made significant achievements during the past decade towards building well-equipped PHC organizations to better meet public expectations in the field of organizational resources and geographical accessibility. However, there are still challenges to improve service delivery and clinical practice, especially in quality, integration, and management, which have also been highlighted in many other low-and-middle income countries. In recent years, China has made efforts to address these challenges, mainly through building a family doctor contract service model and tiered health-care delivery system with bidirectional referral mechanisms [13, 14]. However, challenges remain in translating these policy efforts into daily practice among front-line PHC organizations. Our framework provides a comprehensive list of important elements to consider in implementing solutions at the organizational level. It is equally important to take into account the lessons learned and innovative solutions from international experiences of primary care reform, such as the Family Health Teams in Ontario [50] and Family Medicine Groups in Quebec [51].

The main strength of this research is that it reflects patient preferences regarding high performing PHC organizations. Our sample was diverse and included participants from a vast geographical area (eastern, middle, and western China) and participants of different genders, age, and health insurance types. Second, we used an established analytical framework—the Classification System of PHC Organizational Attributes, which was developed based on a comprehensive scanning study of literature on the organization of PHC. This scientific framework was used to organize the preliminary structure of the domains and attributes while considering the primary care context in China. However, the following limitations should also be considered. There is a high demand for health care among the rural population. Their expectations were not explored in this study. Because of the differences in primary care systems and health insurance programs between urban and rural areas in China, the transferability of our results is limited. Future studies could investigate the preferences of rural populations regarding PHC organizations and compare them with those of urban populations. Finally, translating data from Mandarin into English may have resulted in some linguistic inconsistencies.

## Conclusions

In summary, we generated a profile of high performing PHC organizations from the Chinese patient perspective, that includes five domains: organizational resources, general features, service provision and clinical practices, quality and cost, and organizational structure. Organizational resources, service delivery and clinical practice were the domains that were most valued by the Chinese patients. Meanwhile, the participants also had strong expectation of geographical accessibility, high quality of care as well as efficient organizational structure. These organizational elements should be reflected in further reform efforts in order to build high performing PHC organizations.

## Data Availability

The data that support the findings of this study are available from the corresponding author upon reasonable request.
